# Mobilized peripheral blood: an updated perspective

**DOI:** 10.12688/f1000research.21129.1

**Published:** 2019-12-20

**Authors:** Darja Karpova, Michael P. Rettig, John F. DiPersio

**Affiliations:** 1 Division of Stem Cells and Cancer, German Cancer Research Center (DKFZ) and DKFZ-ZMBH Alliance, Heidelberg, 69120, Germany; 2 Division of Oncology, Department of Medicine, Washington University School of Medicine,, St. Louis, Missouri, 63110, USA

**Keywords:** Mobilization, Hematopoietic stem and progenitor cells, G-CSF, CXCR4, VLA4, CXCR2, BM niche, Chemosensitization, Conditioning, Gene therapy

## Abstract

Enforced egress of hematopoietic stem cells (HSCs) out of the bone marrow (BM) into the peripheral circulation, termed mobilization, has come a long way since its discovery over four decades ago. Mobilization research continues to be driven by the need to optimize the regimen currently available in the clinic with regard to pharmacokinetic and pharmacodynamic profile, costs, and donor convenience. In this review, we describe the most recent findings in the field and how we anticipate them to affect the development of mobilization strategies in the future. Furthermore, the significance of mobilization beyond HSC collection, i.e. for chemosensitization, conditioning, and gene therapy as well as a means to study the interactions between HSCs and their BM microenvironment, is reviewed. Open questions, controversies, and the potential impact of recent technical progress on mobilization research are also highlighted.

## Introduction

Discovered by pure chance in patients recovering from chemotherapy almost 45 years ago ^1^, the phenomenon of hematopoietic stem cell (HSC) mobilization has transformed the clinical practice of HSC transplantation ^2^. It has further extended to indications beyond HSC collection, including mobilization-based chemosensitization, conditioning, and gene therapeutic approaches, which are areas of intensive research. Better understanding of the pathways governing HSC trafficking can provide important insights into how stem cell localization within the bone marrow (BM) is regulated, which explains a continued need for basic research on mobilization to define the underlying molecular and cellular mechanisms.

**Figure 1.**
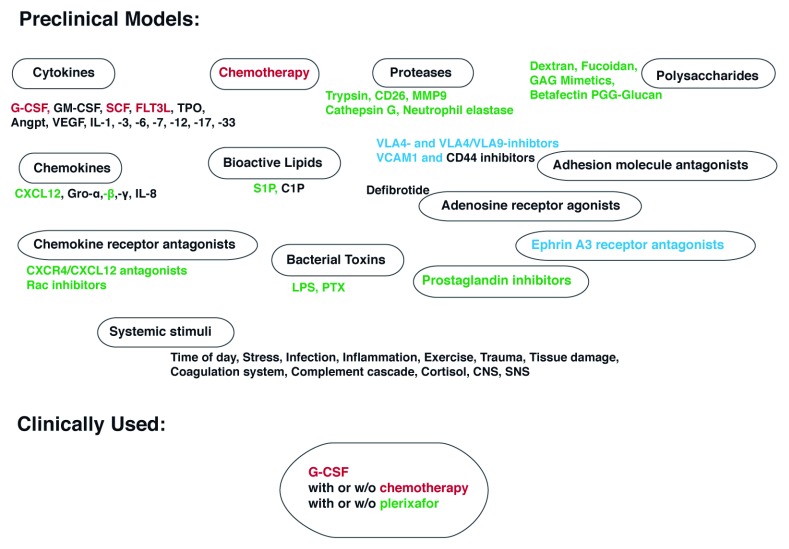
Mobilization stimuli. A wide variety of stimuli that lead to increased numbers of circulating hematopoietic stem cells (HSCs) have been identified, including but not limited to growth factors (cytokines ^17^, granulocyte colony-stimulating factor [G-CSF] ^19, 38^, granulocyte-macrophage colony-stimulating factor [GM-CSF] ^20^, stem cell factor [SCF] ^18, 39, 40^, FLT3 ligand [FLT3L] ^40– 42^, thrombopoietin [TPO] ^15, 16, 40^, angiopoietin [Angpt] ^43, 44^, vascular endothelial growth factor [VEGF] ^43– 45^, and interleukins [ILs] -1 ^46^, -3 ^47, 48^, -6 ^49^, -7 ^50^, -12 ^51^, -17 ^52^, and -33 ^53, 54^), chemotherapy-induced myeloid rebound ^1, 23, 55^, chemokines (CXCL12 and analogs ^27, 56^, Gro-α ^57^, -β ^26, 58, 57, 59^, and -γ ^57^, and IL-8 ^24, 25, 60^), chemokine receptor antagonists (CXCR4 antagonists ^61, 62, 63^ and inhibitors of the intracellular mediator of CXCR4 signaling, the small Rho GTPase Rac1 ^64, 65^), bioactive lipids (sphingosine-1 phosphate [S1P] ^66, 67^ and ceramide-1 phosphate [C1P] ^66, 67^) and bacterial toxins ^36, 68, 69^ (lipopolysaccharide [LPS] ^37, 70^ and pertussis toxin [PTX] ^71– 74^), proteases (trypsin ^75^, matrix metalloprotease 9 [MMP9] ^58, 76^, CD26 ^77, 78^, cathepsin G ^79^, and neutrophil elastase ^79^) and adenosine receptor agonists (defibrotide) ^80, 81^, inhibitors of adhesive cell interactions ^82^ (VLA4 ^28, 29, 83^ and VLA4/VLA9 ^84^ inhibitors, VCAM1 ^85^, and CD44 ^86, 87^ blockers) and ephrin A3 receptor antagonists ^88^, polymeric sugar molecules (dextran ^89^, fucoidan ^89, 90^, Betafectin PGG-Glucan ^91^, and glycosaminoglycan [GAG] mimetics ^92^), and prostaglandin inhibitors ^93^. For the majority of the listed stimuli, a direct or indirect targeting of CXCR4 (green) or VLA4 (blue) signaling or both (red) has been documented. On the systemic level, the time of day ^10, 11, 94^, stress ^95, 96^ and exercise ^97– 100^, trauma and tissue damage ^101– 103^, infection ^104^ and inflammation ^105, 106^, coagulation ^107– 109^ and complement ^110– 113^ cascade along with cortisol ^94, 114^ and the central ^114^ as well as the sympathetic nervous system (SNS) ^115^ have been shown to affect HSC egress out of the bone marrow (BM) into the peripheral blood. In sharp contrast to the diversity of mobilizing agents discovered and tested in preclinical models, only G-CSF alone (healthy donors) or in conjunction with chemotherapy and plerixafor (patients) is being used in the clinic. CNS, central nervous system.

In mammals, the first definitive HSCs arise in several intra and extraembryonic tissues from which they first migrate into the fetal liver ^3, 4^. Following expansion in the fetal liver, HSCs continue their journey towards the BM, where the overwhelming majority of adult HSCs are subsequently found in their unique, specialized environments, the BM niches ^5, 6^. Interestingly, despite the dramatically reduced migratory activity upon BM colonization, a small fraction of adult HSCs can be found in the peripheral circulation at any given time ^7– 9^. Even though random leakiness of BM retention pathways cannot be excluded as a cause, the regularity of this physiological HSC egress ^10– 12^ implies a biological function. The number of HSCs in the circulation at steady state can be substantially augmented by a wide variety of endogenous and exogenous stimuli such as growth factors ^13– 20^, chemotherapy ^1, 21– 23^, chemokines ^24– 27^, chemokine and integrin receptor agonists and antagonists ^28– 31^, bioactive lipids ^32, 33^, exercise ^34, 35^, infection, and inflammation ^36, 37^ ( Figure 1). This enforced egress of HSCs into peripheral blood is called mobilization. While the function of homeostatically circulating HSCs remains enigmatic, pharmacologically induced HSC egress is increasingly used as the preferred strategy to generate grafts for HSC transplantation (HSCT), the only curative therapeutic option for many hematopoietic malignancies as well as non-malignant pathologies. HSCT requires the intravenous infusion of a minimum of 2×10 ^6^ CD34 ^+^ stem cells/kg recipient body weight; however, a dose of 5×10 ^6^ CD34 ^+^ cells/kg is considered preferable for early, consistent, and long-term multilineage engraftment ^116– 118^. Each failure or delay to collect sufficient hematopoietic stem/progenitor cells (HSPCs) to proceed to transplantation extends the time of high-dose chemotherapy and increases the risk of disease progression in cancer patients.

The need to optimize mobilization regimens with regard to their stem cell yield, side effects and risk profile, cost-effectiveness, and availability for different groups of patients, as well as the need to better understand the communication between HSCs and their niche, continues to drive mobilization research. In this review, we discuss how deciphering the events induced by the most commonly used mobilizing agent, granulocyte colony-stimulating factor (G-CSF), led to the development of new mobilization strategies. We highlight the most recent findings and how we envision the newly discovered mobilization approaches will impact mobilization in the clinic. Alternative applications for mobilization are also reviewed. Lastly, we identify open questions and controversies, prospective directions, and how recent technical advances can be implemented within mobilization research.

## Current mobilization regimens

G-CSF-mobilized blood is the preferred graft source for virtually all autologous and an increasing majority of allogeneic HSCTs owing to its generally higher stem cell content, reduced rates of graft failure, and better overall survival as compared to the BM ^2, 119, 120^. After 4–5 days of treatment with G-CSF, circulating HSPCs increase an average of 50–100-fold ^121, 122^ as a result of HSPC pool expansion followed by mobilization. The latter is achieved through targeting the two major pathways involved in stem cell retention: chemokine receptor CXCR4- and integrin VLA4-mediated signaling ^79, 123, 124^. Attenuation of these pathways is achieved on the level of gene expression as well as through proteolytic cleavage ^79, 123– 127^. While the role of specific proteases involved in the latter, such as neutrophil elastase, cathepsin G, and MMP9, remains controversial ^128, 129^, the cell surface protease dipeptidyl peptidase 4 (DPP-4, CD26), which cleaves and inactivates the CXCR4 ligand CXCL12, has indeed been shown to be essential for G-CSF-induced mobilization ^77, 78^.

Shortcomings of G-CSF such as the slow mode of action ^17, 130^, side effects, and contraindications ^131, 132^ as well as significant heterogeneity in the mobilization response ^121^ explain the quest for alternative mobilizing agents ^133^. Plerixafor (AMD3100), a small molecule bicyclam CXCR4 antagonist, is FDA approved for autologous stem cell mobilization in non-Hodgkin’s lymphoma and multiple myeloma (MM) ^134, 135^. When combined with G-CSF, plerixafor increases CD34 ^+^ concentration 2–3-fold compared to G-CSF alone ^134, 135^. However, a significant disadvantage of plerixafor is its cost, adding $25,567 per patient compared to G-CSF alone ^136^. Furthermore, up to 24% of patients undergoing autologous stem cell transplantation for lymphoma receiving plerixafor and G-CSF still fail to collect ≥2×10 ^6^ CD34 ^+^ cells/kg in 4 days of apheresis ^134, 135^. A recent economic analysis determined that reducing apheresis by 1 day can potentially decrease medical costs by $6,600 ^137^. Thus improved/alternative mobilizing agents and strategies are needed.

The long-standing view has been that both HSPC expansion and mobilization are necessary for clinically relevant mobilization. In line with this view, mobilization with CXCR4 or VLA4 antagonists alone fails to achieve numbers that would allow their use without G-CSF, despite promising potential in preclinical models ^29, 61, 83, 138– 140^. Very recent findings by our group and others challenge this notion and suggest that efficient recruitment of long-term, serially repopulating HSCs can be accomplished within minutes ^57, 58^. Indeed, CXCR4 or VLA4 blockade, when combined with the stimulation of a different chemokine receptor, CXCR2, results in extremely rapid and potent HSC mobilization in mice with a repopulating capacity similar or even superior to G-CSF-recruited HSCs ^57, 58^. These observations show that major changes in cellular composition or localization are not required for efficient mobilization. They further highlight the existence of different HSC species that, upon disruption of certain adhesive tethers, can egress from the BM very rapidly with kinetics that appear incompatible with a prior requirement for changes in gene expression.

**Figure 2.**
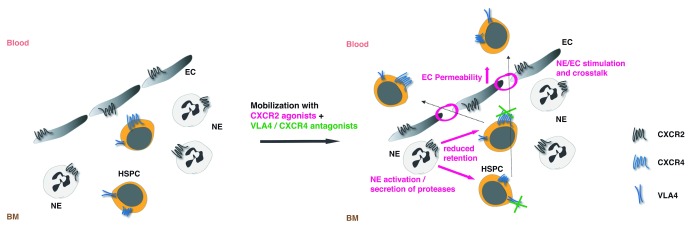
Mobilization priming effects of CXCR2 stimulation. Upon activation of the CXCR2 receptor on the surface of neutrophils (NE) and/or endothelial cells (EC), a reciprocal stimulation of the cells occurs that is critical for the subsequent boost of mobilization with a VLA4 or CXCR4 antagonist. Augmented mobilization appears to be a result of increased permeability of the endothelial layer together with other cell contact mediated or soluble factors derived from CXCR2-stimulated cells that reduce hematopoietic stem/progenitor cell (HSPC) retention. Additional inhibition of the VLA4 or CXCR4 receptor results in efficient targeting of the very primitive, serially repopulating HSPCs retained in the bone marrow (BM) primarily via VLA4 or CXCR4 signaling, respectively. The rapid kinetics of CXCR2 agonist + VLA4 or CXCR4 antagonist-induced mobilization preclude major molecular or cellular changes prior to the BM egress and rather suggest a close proximity of the primitive HSPC fraction to the BM sinusoids.

The key role of the stromal compartment in G-CSF-induced mobilization has long been appreciated ^124, 129, 141^. Following activation of their G-CSF receptor, BM monocytes/macrophages, the most prominent hematopoietic component of the BM stroma ^142– 144^, downregulate several retention molecules, including the major CXCR4 ligand, CXCL12, and several VLA4 ligands by non-hematopoietic stroma, resulting in HSPC egress ^143, 145– 147^. Absence of the G-CSF receptor on monocytes/macrophages abrogates the G-CSF-induced mobilization response ^143^, whereas the cytokine oncostatin M is thought to mediate communication between monocytes and non-hematopoietic stroma ^145, 148^. Interestingly, rapid mobilizing agents in general, and chemokines and chemokine receptor antagonists in particular, are assumed to act on hematopoietic cells directly. Our recent findings challenge this view and suggest a critical contribution of stromal (endothelial) CXCR2 targeting upon rapid HSC mobilization by the combination of the CXCR2 ligand truncated Gro-beta (tGro-β) and a VLA4 antagonist ^57^. Since CXCR2 is absent from the HSPC surface ^25, 26^, CXCR2-expressing neutrophils have long been assumed to be the responding cell. Upon stimulation with CXCR2 agonists, neutrophils release proteases that cut adhesive interactions between HSPCs and their niche ^60, 76, 149, 150^. Yet CXCR2 within stroma was sufficient to induce mobilization with tGro-β and a VLA4 antagonist ^57^, pointing towards changes in endothelial layer permeability ^151^ as well as crosstalk between endothelia and neutrophils as the “priming” events triggered by CXCR2 activation that boost VLA4 antagonist-induced HSPC egress ( Figure 2).

## Development of alternative HSPC mobilization regimens and grafts

As we continue to learn about the events and mechanisms regulating HSPC egress, we approach the ultimate goal of developing an “optimal” mobilization strategy to collect sufficient numbers of primitive stem cells with superior properties within a day or two. In contrast to the days when clinical observations determined the applicability of a mobilization approach, educated and targeted designs are becoming the basis for the clinical development of mobilizing agents. In addition to the quantity and fitness of the HSPCs, as reflected in their engraftment capacity, the immunogenic properties of the graft (i.e. the graft-versus-host disease [GvHD] profile) are an important feature requiring optimization, potentially at the cost of stem cell numbers. For example, a substantially reduced incidence of GvHD is observed upon transplantation of CXCR4 antagonist-mobilized grafts, possibly due to co-mobilization of a specific population of dendritic cells (DCs) with immunomodulatory properties, plasmacytoid DCs ^140, 152, 153^. Along the same lines, grafts mobilized with pegylated G-CSF were superior to standard G-CSF in that they were associated with less GvHD ^154, 155^ while graft-versus-leukemia (GvL) effects were improved through mobilization of invariant natural killer T (iNKT) cells ^155^. Susceptibility of the mobilized HSPCs to further molecular manipulation, e.g. using gene therapy, is another important criterion when defining the “optimal” mobilization strategy. Lastly, if proven to be suitable for mobilized HSPCs, recently described methods for *ex vivo* expansion of HSCs ^156^ are expected to shift the emphasis on HSPC quality over quantity even further.

Studies with CXCR4 and VLA4 antagonists, tested in VLA4 and CXCR4 knockout mice, respectively, implied an independence between the two axes ^139, 157, 158^. This suggests that subsets of HSPCs are being retained in the BM by either CXCR4 or VLA4. Combined with the knowledge of the complexity and multiplicity of events induced in the course of G-CSF mobilization ^129, 133^, co-existence of these (and possibly other) functionally distinct HSPC populations suggests combinatorial mobilization approaches as the best alternatives to G-CSF. Thus, the small molecule Me6TREN reportedly inhibits CXCR4 and VLA4 signaling simultaneously, possibly through upregulation of the protease MMP9 ^159^. However, given the controversy regarding the role of MMP9 for mobilization ^128^, other approaches should be explored. In addition to cell-intrinsic HSPC retention pathways, disruption of endothelial layer integrity, along with the endothelial cell activation and subsequent crosstalk between endothelial and mature hematopoietic cells, should be included in designing “optimal” mobilization. Recent data suggest that Viagra (sildenafil citrate), a phosphodiesterase type 5 (PDE5) inhibitor which blocks the degradation of cyclic GMP in the smooth muscle cells lining blood vessels, resulting in vasodilation, can synergize with plerixafor to rapidly mobilize stem cells in mice ^160^.

Various techniques for *ex vivo* graft manipulation (e.g. T cell depletion and CD34 enrichment ^161– 164^) have been developed that entail extended periods during which the HSPCs stay outside of their natural environment and therefore, unsurprisingly, exhibit reduced stem cell capacity ^165, 166^. From further in-depth analyses of differentially mobilized blood (see below), we expect to learn not only how to target specific HSPC populations but also how to mobilize HSPCs without a concurrent mobilization of mature cells, T-cells in particular. In general, *a priori* cell type-specific targeting remains challenging because of the high conservation of migratory and retention pathways between different hematopoietic cell types. Nevertheless, selective HSPC mobilization represents an intriguing goal that would help reduce additional graft manipulation.

## Mobilization beyond stem cell collection

### Chemosensitization

In addition to supplying HSPCs with the factors required for their normal development, the BM microenvironment is also a refuge for malignant cells, allowing them to escape cytotoxic therapies and cause disease relapse ^167, 168^. This provides a rationale for targeting the interactions between tumor cells and the BM, with the goal of sensitizing them to therapy. Pathways responsible for the anchorage and survival of malignant cells and resistance to chemotherapy largely overlap with those of normal HSPCs ^168, 169^. Accordingly, blockade of CXCR4 and VLA4 signaling and/or G-CSF was tested in conjunction with chemotherapy in pre-clinical models of acute myeloid leukemia (AML ^170– 173^), acute ^174, 175^ and chronic ^176^ lymphoid leukemia, and MM ^177^. Moreover, the FDA-approved CXCR4 antagonist plerixafor has been tested as a chemosensitizing agent alone and in combination with G-CSF in patients with relapsed AML ^178, 179^. While the mobilizing capacity varied substantially, an overall benefit from adding mobilizing agent(s) to chemotherapy has been reported, prolonging survival and decreasing tumor burden ^170, 172, 177, 180^ or even eradicating disease ^175^. The benefits of this approach in AML and other hematologic malignancies, in spite of these preclinical as well as early clinical studies, remain both unclear and controversial.

### Conditioning

As HSPCs are pharmacologically driven from the BM into circulation, the temporarily unoccupied spaces (niches) in theory become available to new cells, e.g. the HSPCs introduced into a mobilized recipient during transplantation. The utility of mobilization for non-cytotoxic and on-target conditioning prior to HSCT is supported by the fact that mobilized cells return to the BM after spending some time in peripheral circulation, as shown in studies of parabiotic mice ^181^. Yet virtually all attempts at mobilization alone for conditioning of an adult host before HSCT have been unsuccessful (Karpova and Rettig, unpublished data). It is unclear whether the reason is that the cells introduced exogenously are inherently disadvantaged (less fit?) compared with endogenously circulating HSPCs or whether the mobilizing agent interferes with the repopulating capacity of the transplanted cells. An intriguing alternative explanation is that owing to targeting/recruitment of a specific population during the mobilization process, and by extension because of emptying of very specific niches, only HSPCs mobilized with the same mobilizing regimen are able to engraft BM niches that become available. Interestingly, since BM- or fetal liver-derived HSPCs have been used to engraft mobilized recipients (Karpova and Rettig, unpublished data), the possibility that a qualitative rather than quantitative approach might lead to successful, persistent engraftment is untested. Given recent reports of successful conditioning using antibody–drug conjugates targeting the pan leukocyte marker CD45 ^182^ and the CD117-targeting antibodies ^183– 185^, or a cocktail of monoclonal antibodies depleting CD47-expressing cells along with T cells, NK cells, and HSPCs ^186^, mobilization-based conditioning may not be a promising approach in postnatal recipients. However, fetal HSPC mobilization with a VLA4 antagonist followed by *in utero* HSCT results in increased donor HSC homing to the fetal liver and enhanced long-term allogeneic engraftment in mice ^187^. Therefore, mobilization-based conditioning regimens might be applicable for *in utero* HSCT.

### Gene therapy

Manipulating HSCs to correct mutations that cause inherited diseases of the hematopoietic system such as sickle cell anemia and beta-thalassemia represents a potential cure, with recent advances in gene therapeutic approaches (CRISPR-Cas9, TALEN, and ZFN) ^188– 193^ allowing sustainable correction of the genetic defects. Autologous HSCT is the method of choice in this setting, whereby instead of extracting HSCs for subsequent *ex vivo* manipulation, stem cells are mobilized into the circulation and subjected to gene therapy *in situ* ^194– 196^. As discussed above, HSC collection and *ex vivo* editing inevitably leads to a diminished stem cell capacity, which can be avoided by editing the cells in the peripheral blood. Proof of principle for mobilization-based gene therapy was reported following mobilization with G-CSF plus a CXCR4 antagonist, with sustained expression of the introduced transgene over a period of 5 months ^196^. We believe this approach should be developed further, e.g. by using it in combination with mobilization strategies to preferentially recruit stem cells with superior repopulating capacity into the circulation. In addition, these cells may be more susceptible to therapeutic gene editing. Apart from its obvious therapeutic benefit, this approach might become useful for studying functional differences between HSCs that have been mobilized into the circulation and returned to the BM and HSCs that remain in the BM niche.

### Biology of the hematopoietic niche

The discovery of compounds and pathways that enable HSPC displacement from the niche has provided important insights into the regulation of HSPC trafficking and maintenance. For example, detailed analysis of the mechanisms underlying G-CSF-induced mobilization was indispensable for establishing monocytes/macrophages as crucial components of the BM niche and understanding their crosstalk with the non-hematopoietic stroma ^143, 145– 147^. More recent studies demonstrated that bone marrow dendritic cells regulate endothelial cell function in part through CXCR2 signaling, resulting in HSPC mobilization and loss of bone marrow macrophages ^151^. Similarly, studies with the CXCR2 ligand tGro-β disclosed the role of another mature leukocyte population, neutrophils, in HSPC trafficking ^57, 58, 76^. Together with the observation that the circadian release of HSPCs into the circulation is synchronized by daily return of aged neutrophils from the circulation into the BM ^197^, these findings implicate neutrophils as critical mediators of HSPC localization at steady state and upon enforced egress. On the molecular level, the recognition that all physiological, pathological, and pharmacological mobilization stimuli described to date interfere with VLA4 or CXCR4 signaling or both ( Figure 1) highlights the key roles of these two pathways in HSPC trafficking.

## Open questions and future directions

### Homeostatic HSPC trafficking

The physiological function and regulation of daily HSPC egress remain elusive. We know that, similar to their mature counterparts, the release of HSPCs from the BM follows a circadian rhythm ^11, 94, 198^, with sympathetic nervous system-derived adrenergic signals acting through beta(3)-adrenergic receptors on BM stroma to downregulate CXCL12 signaling (and therefore retention ^10, 11^). While we understand the purpose of migration of more differentiated hematopoietic cells out of the BM to undergo maturation, encounter antigens, proliferate, etc., the role(s) of HSPCs found in blood and other peripheral tissues is speculative. Exchange between different parts of the hematopoietic system has been suggested to be mediated by homeostatic HSPC migration ^7, 133^ and is supported by observations from parabiotic ^9, 199, 200^ as well as partially irradiated ^201, 202^ mice. An alternative explanation implies a possible immune surveillance function of HSPCs that have also been found in the lymphoid system ^203^ and non-hematopoietic tissues with crucial immune functions such as the intestine ^204^. Furthermore, expression of MHC class II molecules, otherwise restricted to professional antigen-presenting cells, has been detected in HSPCs ^205, 206^.

### Prediction of HSPC mobilization success and failure

With regard to inadequate HSPC mobilization, one must distinguish between disease- and/or treatment-associated failure and failure of G-CSF mobilization in healthy donors. Of note, less than 1% of “healthy allogeneic donors” fail to collect an optimal (5×10 ^6^ CD34/kg) or minimal (2×10 ^6^ CD34/kg) amount of CD34 ^+^ cells after a standard 4–5-day regimen of G-CSF mobilization ^133^. This relatively uncommon event may represent the extreme heterogeneity of HSPC mobilization seen among the general population with no known medical conditions or prior exposure to chemotherapy or radiation. Thus, CD34 counts between 5 and 500 cells per µl blood have been reported ^121^. The general consensus, derived from studies of poor- and well-mobilizing mouse strains ^207, 208^, as well as repetitive mobilization of healthy donors ^121, 209^, is that genetic factors determine the mobilization response in healthy individuals. However, single nucleotide polymorphisms in any of the obvious candidate genes (including CXCL12 ^210^, VCAM1 ^211, 212^, and CD44 ^212, 213^) do not correlate with mobilization efficacy in response to G-CSF or plerixafor in larger population studies ^133, 214, 215^. Knowledge of high or low HSPC mobilization potential could be translated into donor screening prior to mobilization to help predict response and potentially to guide the best mobilization strategy.

The probability of mobilization failure in patients directly correlates with the amount and extent of prior cytotoxic exposure ^216– 218^. Other clinical and demographic features of normal donors and patients undergoing autologous stem cell mobilization that predict poor mobilization include age ^217, 219^, resting platelet counts ^217, 220^, and a history of diabetes mellitus ^221^. Failure rates have decreased since the CXCR4 antagonist plerixafor was approved for the mobilization of HSPCs for MM and non-Hodgkin lymphoma patients when given in conjunction with G-CSF ^133– 135^. However, given that the diminished mobilization response results from the substantially reduced HSPC pool in these patients ^222^, approaches that both potently expand HSPCs and induce their egress into the circulation are needed. We recently reported that the administration of CXCR4 antagonists to mice by subcutaneous continuous infusion for 1–4 weeks induces a robust mobilization response that significantly exceeds the mobilization achieved with bolus drug injections and was 25–50-fold greater than a 5-day course of G-CSF ^158^. Moreover, continuous infusion of CXCR4 inhibitors leads to a two- to four-fold expansion of the HSPC pool in the bone marrow that exhibits a distinct repopulating advantage when tested in serial competitive transplantation experiments. Similarly, others have shown that the FMS-like tyrosine kinase 3 ligand (Flt3L) stimulates the expansion and mobilization of HSPCs in animals and humans ^223– 225^.

### Profiling of differentially mobilized blood

Gene expression profiling of the HSPC populations mobilized with different agents has been performed, mostly using microarrays ^57, 226^. Not unexpectedly, increased relative expression of genes involved in lymphoid development were detected in grafts mobilized with a CXCR4 antagonist alone and in conjunction with G-CSF as compared to G-CSF alone ^226^. A closer similarity between BM-resident and CXCR4 antagonist-mobilized HPSCs as compared to BM-resident versus G-CSF-mobilized HSPCs had been proposed to be due to the fast kinetics of CXCR4 antagonist-induced mobilization. However, the opposite was detected when comparing the three, with BM-resident HSPCs showing a profile much closer to that of G-CSF-mobilized HSPCs ^57^. It would appear from these studies that CXCR4 disruption recruits a specific rather than representative fraction of the BM HSPCs. By contrast, VLA4 antagonist + tGro-β-mobilized HSPCs have a profile very similar to that of BM-resident as well as G-CSF-mobilized HSPCs, indicating that rapid kinetics of mobilization can indeed be associated with an HSPC profile closely related to BM-resident HSPCs ^57^.

Single cell RNA sequencing (scRNA-seq) is currently revolutionizing the field of hematopoietic cancer research by defining the heterogeneity of malignant cells and the supporting network of non-malignant cells ^227– 230^. Naturally, scRNA-seq analysis and comparison of differentially mobilized HSPCs will provide key insights and may strengthen the notion that they are comprised of different HSPC subsets rather than representing a homogenous population with an overall altered expression profile depending on the agent used. Moreover, we anticipate that concurrent analysis of peripheral blood and BM HSPC compartments will shed new light on the unique identity and specific origin of mobilized cells that respond to specific mobilizing agents. In general, single cell characterization of mobilized blood/HSPCs is expected to be particularly informative with rapid-mobilizing agents, where the kinetics of the cell recruitment would not allow for major changes of the cell identity or localization prior to BM egress.

Interestingly, despite the more elaborate isolation process for non-hematopoietic as opposed to hematopoietic cells, a detailed characterization of the stromal populations using single cell approaches has been published already ^231– 233^. However, the contribution of the newly identified populations to HSPC retention remains unexplored. Ultimately, simultaneous analysis of stroma and HSPCs based on their proximity using spatial transcriptomics promises to reveal potentially unique relationships between certain stromal and hematopoietic cell types and thereby define the biological roles of the long-argued diversity within the hematopoietic niche.

## Summary

Pharmacologically induced egress of HSPCs from the BM has become an indispensable tool in HSCT with all autologous and over 80% of allogeneic transplants performed with mobilized blood. Mechanistic insights gained from studying the complex chain of events induced during mobilization with G-CSF have paved the way for the rational design of alternative mobilization approaches without the inherent shortcomings of G-CSF such as slow mode of action and side effects. Similar to the recruitment of healthy HSPCs into the circulation, targeting of various BM retention pathways has been explored as a means to sensitize leukemic cells and thereby improve the efficacy of chemotherapy. Moreover, mobilization of HSPCs as a non-cytotoxic conditioning strategy as well as for gene therapy represents two other applications of mobilization beyond mere collection of a stem cell graft. Understanding the physiological function of homeostatic HSPC trafficking and identifying the genetic determinants of mobilization efficiency, along with characterizing the differentially mobilized HSPC populations on a single cell level, represent some of the directions of future mobilization research.
